# Effects of live and video simulation on clinical reasoning performance and reflection

**DOI:** 10.1186/s41077-020-00133-1

**Published:** 2020-07-31

**Authors:** Timothy J. Cleary, Alexis Battista, Abigail Konopasky, Divya Ramani, Steven J. Durning, Anthony R. Artino

**Affiliations:** 1 Rutgers, The State University of New Jersey, New Brunswick, USA; 2 Center for Health Professions Education, F. Edward Hebert School of Medicine, Uniformed Services University of the Health Sciences, 4301 Jones Bridge Road, Bethesda, MD 20814-4712 USA; 3 Department of Health, Human Function, and Rehabilitation Sciences, The George Washington University School of Medicine and Health Sciences, Washington, USA

**Keywords:** Simulation, Medical simulation, Healthcare simulation scenario-based simulation, Video-based simulation, Self-regulated learning microanalysis, Functional linguistics, Linguistic analysis, Cognitive processes, Metacognitive processes

## Abstract

**Introduction:**

In recent years, researchers have recognized the need to examine the relative effectiveness of different simulation approaches and the experiences of physicians operating within such environments. The current study experimentally examined the reflective judgments, cognitive processing, and clinical reasoning performance of physicians across live and video simulation environments.

**Methods:**

Thirty-eight physicians were randomly assigned to a live scenario or video case condition. Both conditions encompassed two components: (a) patient encounter and (b) video reflection activity. Following the condition-specific patient encounter (i.e., live scenario or video), the participants completed a Post Encounter Form (PEF), microanalytic questions, and a mental effort question. Participants were then instructed to re-watch the video (i.e., video condition) or a video recording of their live patient encounter (i.e., live scenario) while thinking aloud about how they came to the diagnosis and management plan.

**Results:**

Although significant differences did not emerge across all measures, physicians in the live scenario condition exhibited superior performance in clinical reasoning (i.e., PEF) and a distinct profile of reflective judgments and cognitive processing. Generally, the live condition participants focused more attention on aspects of the clinical reasoning process and demonstrated higher level cognitive processing than the video group.

**Conclusions:**

The current study sheds light on the differential effects of live scenario and video simulation approaches. Physicians who engaged in live scenario simulations outperformed and showed a distinct pattern of cognitive reactions and judgments compared to physicians who practiced their clinical reasoning via video simulation. Additionally, the current study points to the potential advantages of video self-reflection following live scenarios while also shedding some light on the debate regarding whether video-guided reflection, specifically, is advantageous. The utility of context-specific, micro-level assessments that incorporate multiple methods as physicians complete different parts of clinical tasks is also discussed.

## Introduction

Clinical reasoning—the gathering and integration of clinical information combined with medical knowledge to generate a diagnosis and treatment plan—is a complex and challenging endeavor requiring extensive practice to reach proficiency [1, 2]. Even among physicians with many years of experience, diagnostic errors continue to be a problem, accounting for approximately 10% of patient deaths and contributing to other issues, such as delays in diagnosis and treatment and medication errors [3, 4].

Given the need to enhance clinical reasoning proficiency, there has been increased attention on learning methods to optimize these abilities. Common approaches include lectures, case-based learning, clinical case discussions, workplace learning, and simulation-based learning [5]. Simulation-based formats, which include virtual patients, pre-recorded videos (i.e., vignettes depicting a doctor-patient encounter [6]), and live scenarios (i.e., structured narrative embedded within a simulated clinical setting) [7, 8] have increased in popularity over the years. Their popularity has grown, in part, because they closely mirror authentic, clinical settings and patient-provider interactions [6], afford opportunities to practice myriad clinical activities in different contexts [9], and enable extensive opportunities for reflection [10, 11].

Although some researchers have examined the individual effects of traditional (e.g., paper cases) and simulation learning environments [12, 13], very few have examined the *relative effectiveness* of such approaches for enhancing clinical reasoning abilities [14]. Further, learning effectiveness research has typically focused on performance *outcomes* (e.g., diagnoses and direct observation in clinical or simulated settings) rather than the *processes* and overall *experiences* of medical professionals during clinical activities. Given these gaps, we experimentally examined the differential effects of two simulation learning environments (i.e., video and live scenario) across performance outcomes as well as the task-specific perceptions, cognitive reactions, and reflective judgments of medical professionals during clinical reasoning.

### Clinical reasoning as complex and situated

Although clinical reasoning is often conceptualized as an end *product*, Ilgen, Eva, and Regehr argue that it can also be viewed as a complex, dynamic, and often uncertain *process* of meaning making [15]. They argue that the skillful deployment and completion of clinical reasoning tasks shift according to the case and context, painting a complex and situation-specific (situated) picture of clinical reasoning [15]. Beyond the complexity of the clinical reasoning tasks themselves, there is a developing literature on *contextual factors*—common features of clinical practice (e.g., patient frustration, interruptions, and language barriers) that typically are not used to establish the correct diagnosis [16–18]. Based on recent research [19, 20] and the theoretical proposition that knowing is bound to activity, social norms, environment, and cultural factors [21], the presence of contextual factors can lead physicians to think about and react to different aspects of a case. Differences in situation-specific perceptions and the metacognitive reactions to contextual factors can greatly alter the quality or accuracy of physicians’ diagnostic and management reasoning [18, 22].

### Clinical reasoning and simulation-based learning environments

A variety of learning environments have been used to teach and assess clinical reasoning abilities and often emphasize differences in what is learned. For example, case-based learning and virtual patients emphasize the development of cognitive processes (i.e., interpretation of findings and hypothesis generation), whereas morbidity and mortality rounds and small group coaching place more of an emphasis on metacognition (i.e., monitoring and reflecting on one’s own thought processes) and educational strategies [23]. While all such approaches can support both cognitive and metacognitive skills to some degree, simulation-based learning environments are particularly well suited to address both [10, 11, 24]. Moreover, several studies highlight how post-simulation reflection can support participants’ clinical reasoning as they consider the meaning of their actions and experiences and scrutinize personal assumptions [25, 26].

All simulation environments overlap in terms of participant experiences. When comparing live scenarios and video case formats, both situate the clinical encounter in a fictitious, yet realistic setting depicting a provider-patient interaction [9, 27]. They also emphasize a sequential approach to presenting information (i.e., starting with a greeting, followed by a patient interview) and encourage participants to identify relevant clinical information, identify hypotheses, and solve a clinical problem [27, 28]. However, video cases and live scenarios can be distinguished in terms of duration, efficiency, and complexity of social interactions.

Video cases are quite popular, in part, because of their efficiency and accessibility. Participants are asked to view a pre-recorded provider-patient encounter that has a fixed and often short delivery time. The sequence of case content (e.g., interview, physician exam maneuvers, and lab results [27]) is pre-determined, so participants cannot influence aspects of the encounter. Conversely, live scenario-based simulations are more complicated and difficult to use, in part, because of the need for specially trained individuals (e.g., standardized patients, and simulationists) and the significant time required for design and implementation [29, 30]. Live scenarios also tend to be more intensive in that participants need to engage in complex, clinical activities (e.g., structured interventions such as focused assessment) while concurrently determining optimal ways to sequence these activities, an experience characterized by high levels of autonomy, agency, and cognitive demands [7]. Live scenarios can also be more unpredictable in terms of the duration of the patient encounter and the nature of the physician or patient responses [7].

These structural distinctions are not perfunctory, as they have the potential to influence the nature of the clinical reasoning processes used by medical professionals as well as their subjective reactions. Further, although researchers have examined the influence of different simulation approaches used to teach and evaluate clinical reasoning, such as live scenarios and videos, systematic and direct comparisons of these approaches remain limited [9, 14, 31–33]. Broadly speaking, the literature is mixed regarding the relative superiority of any given approach. For example, while Durning and colleagues reported no differences in clinical reasoning performance across standardized patient case, video case, and paper case formats [34], LaRochelle and colleagues observed that standardized patient cases and video cases were superior to paper cases, but only for certain subject areas [14].

### Assessing processes during clinical reasoning

Early efforts to examine clinical reasoning processes emphasized behavioral observations and think-aloud protocols [35–37]. This early research helped establish a foundation for understanding the types of actions comprising the clinical reasoning process, such as interviewing, physical assessment, and testing hypotheses. While think-aloud protocols continue to be used within medical education [38], there have been recent attempts to apply unique analytic approaches, such as linguistic analysis, to interpret think-aloud data [20, 39]. One promising tool for understanding the process of clinical reasoning is automated coding of linguistic markers of cognitive processing using the Linguistic Inquiry and Word Count (LIWC) software [40, 41]. One set of LIWC markers is related to cognitive activity along with six dimensions: *insight* (e.g., think and know), *cause* (e.g., because and effect), *discrepancy* (e.g., should and would), *tentativeness* (e.g., maybe and perhaps), *certainty* (e.g., always and never), and *differentiation* (e.g., but and else) [42]. Frequency of these “cognitive processing” words corresponds with higher mental effort and greater focus on tasks like discerning, determining causal relations, and differentiating [43].

Self-regulated learning (SRL) microanalytic protocols have also been used to assess medical professionals’ cognitive and regulatory processes (e.g., planning, monitoring, and evaluative judgments during clinical reasoning) [38, 44–47]. These assessment protocols consist of contextualized questions directly targeting specific regulatory processes (e.g., monitoring and adaptive inferences) that are administered as individuals complete a target activity. Grounded in a social-cognitive perspective that SRL is a dynamic, three-phase cyclical process (i.e., forethought, performance, and reflection), SRL microanalytic protocols are able to assess how individuals strategically approach a task and set goals (i.e., forethought phase), control and monitor task completion (i.e., performance phase), and evaluate and reflect on performance (i.e., self-reflection phase) [46, 48].

## Purposes

The purposes of the current study were to examine the cognitive and regulatory experiences of physicians as they engaged in a simulated outpatient visit, and to explore performance differences across two simulated experiences. Given the paucity of studies directly comparing simulation approaches and the general lack of attention targeting how physicians think and react in such situations, we utilized a multi-method assessment approach to address two broad research questions.

- Are there differences in clinical reasoning performance across video case and live scenario conditions?
- Do physicians participating in live scenarios exhibit different reflective judgments (i.e., perceived challenges and adaptive inferences) and cognitive processing than those in the video case condition?

Given the key structural and format distinctions between live and video case scenarios, we predicted that the experiences and thought processes of the two conditions would differ. Although we could make not a priori predications regarding the specific *types* of cognitive or regulatory group distinctions, we postulated that physicians in the live condition would exhibit a more adaptive profile; that is, they would focus more directly on the clinical reasoning process and the management and integration of data.

We also predicted that physicians in the live scenario group would exhibit better overall clinical performance. Although prior research on the effects of learning environments conveys null or mixed effects, much of this research has used broad-based outcomes to examine performance differences (e.g., objective clinical structured exam [OSCE]). We anticipated that group performance differences would emerge with the use of a contextualized post-encounter form (PEF) that was directly linked with the case used in the provider-patient encounter.

## Method

### Sample

This study was conducted at three different military facilities across the USA with 38 military family medicine, internal medicine, and surgery physicians. The three facilities are educational sites for the Uniformed Services University of the Health Sciences and represent regional tertiary referral centers of similar size for the military population. Physicians within the Military Health System frequently rotate among these and other hospitals. Recruitment efforts included presentations during specialty department (e.g., internal medicine and general surgery) meetings, grand rounds and educational conference sessions, simulation bootcamp sessions, and targeted email campaigns using department lists following department head approval. Recruitment efforts were conducted by research associates.

### Design and procedures

The current study was conducted as part of an investigation funded by the Congressionally Directed Medical Research Program (NH83382416) that sought to broadly examine (a) the effects of contextual factors across diagnosis type and (b) differences in simulation approaches related to clinical reasoning. The current study is aligned with the latter objective and includes data that have not previously been published.

Participants were randomly assigned to either the live scenario or video case group. Before beginning the simulated activity, participants completed an informed consent document and a brief pre-study questionnaire. They were then provided a general overview of the study requirements and expectations [49] and were given think-aloud instruction and practice opportunities, which were scripted for consistency (see Additional file 1). Following these preliminary steps, participants began the patient encounter (i.e., live scenario or video simulation). The simulation activity encompassed two components: (a) patient encounter and completion of the PEF and (b) video think-aloud reflection on the patient encounter.

#### Patient encounter and PEF

In the broader research project, all participants were asked to engage in either two live scenarios or two video cases (set in an outpatient clinical setting). Regardless of simulation modality, all participants completed the PEF for the two cases (i.e., new onset angina and new onset diabetes). The chief complaint, and case content for each case was identical for both conditions (e.g., identical presenting symptoms, language, and gestures to represent those symptoms). Trained simulated participants portrayed the patient in both live and video conditions. Participants were advised that the scenario would run in real time, and that they were to treat the encounter as if it were an actual clinical encounter. The videos portrayed a clinical interview, a brief physical exam, and still screens of laboratory findings (in this order). Participants in the live condition were allowed up to 15 min to complete the case while the video cases were shorter, running approximately 5 min per video. Following each live scenario or video, participants in both conditions were allowed up to 20 min to complete each PEF. Participants were then administered SRL microanalytic and mental effort questions.

#### Think-aloud reflection on patient encounter

Following completion of the first scenario or video case and PEF, participants were instructed to either re-watch the video (i.e., video condition) or to watch a video recording of their own performance (i.e., live scenario condition). Physicians in both conditions received identical instructions; that is, to think aloud without making judgments or offering insights regarding how they came to the diagnosis and management plan.

### Measures

#### Clinical performance

A PEF developed and validated in prior research was used to evaluate the quality of participants’ clinical reasoning [50, 51]. It consisted of seven open-ended scored sections (i.e., history questions, exam actions, problem list, differential diagnosis, leading diagnosis, supporting evidence, and management plan). We used a scoring instrument developed in prior research that has exhibited strong inter-rater reliability (kappa = .82–.93 across sections) [50, 51]. An investigator matched free-text responses to the scoring sheet, which stipulated a score of correct (2 points), partially correct (1 point), or incorrect (0 points) for every potential response. These were all reviewed for accuracy by three internists who reviewed them together to reach consensus. These scores were converted to percentage by dividing total number of points received by total possible score (e.g., if a participant gave two pieces of supporting evidence, they would have a total *possible* score of 4). An aggregate PEF score was calculated and showed adequate internal consistency (*α* = .71).

#### Perceived mental effort

Participants were asked to rate the level of mental effort they expended to complete the PEF following the initial patient encounter. The participants were administered the prompt, “Select your invested mental effort as you worked through the post-encounter form”, and then asked to rate their effort using a 10-point Likert scale ranging from 1 (very low mental effort) to 10 (very high mental effort). This single item-measure of cognitive load has been used in prior studies and has been shown to reliably differentiate groups and to correlate with task difficulty and physiologic measures of cognitive load [17, 52, 53].

#### Microanalytic questions

The authors administered two microanalytic questions immediately following the provider-patient encounter: (a) perceived challenges and (b) adaptive inferences. These free-response questions were similar to those used in prior research except for minor wording modifications to reflect the current learning task [44, 54]. Two individuals independently coded the responses from all 38 participants using a previously established coding scheme [50, 51]. The raters discussed all instances of disagreement, and the lead author made final determinations.

#### Perceived challenge

Consistent with microanalysis methodology, a single item was used to examine the perceptions of physicians regarding challenges encountered when completing the PEF to identify the leading diagnosis (“What was the most difficult thing for you when attempting to come up with the leading diagnosis?”). The participants’ responses were coded into one of the following five categories: (a) analysis of data, (b) personal knowledge/skill, (c) lack of case information, (d) no challenge, and (e) other [44] (see Additional file 2). The inter-rater reliability for this measure was robust as indicated by an agreement of 98.2%.

#### Adaptive inferences

A single-item measure was also used to assess the conclusions that the participants made regarding areas to adapt or improve upon when engaged in similar patient encounters (“Is there anything you would do differently when figuring out the leading diagnosis if you watched the video/participated in the scenario again?”) The coding scheme consisted of four broad categories: (a) general clinical tasks (i.e., history, testing, and physical exam), (b) specific clinical reasoning sub-processes (e.g., identifying symptoms, prioritizing symptoms, and integration), (c) none (i.e., no change was needed), and (d) other (see Additional file 2). The inter-rater reliability for this measure was high (94.8%).

### Think alouds

#### Adaptive inferences—linguistic analysis

To assess adaptive inferences, we used two tools from the functional linguistic study of appraisals (i.e., language people use to evaluate themselves and others): *negation* (negative polarity items like *not*) and *modality* (modal verbs of possibility and obligation like *might* and *should*) [39, 55]. Individuals use negation and modality to bring up alternatives to what actually happened. For instance, “I didn’t ask her about her family history” uses negation not only to point out what s/he did not do, but also to infer that there was another, better way to proceed. “I should have asked her about her family history” uses the modal verb *should* with the same purpose. Linguistic markers of negation and modality allow for inferences about participant conclusions regarding the need to change or adapt one’s approach. These markers can reveal how physicians evaluate themselves and others in clinical environments [39, 56, 57], so we adapted it for better understanding the inferences our participants made about what could have been done differently. Three researchers trained in linguistic analysis coded the think-aloud transcripts for modality (e.g., I/He should have asked that) and negation (e.g., I/He didn’t ask that [but perhaps should have]). The inter-rater reliability for this coding was high (81%, based on two authors coding 15% [*n* = 6] of the transcripts). A binary variable was used to indicate the absence or presence of each linguistic marker.

#### Cognitive processing—linguistic analysis

We used the automated software, LIWC, to record the number of individual markers of cognitive processing including insight, cause, discrepancy, tentativeness, certainty, and differentiation in each participant’s transcript. To account for varying lengths of think-aloud transcripts, LIWC automatically reports each variable as a rate of instances per 100 words. Thus, a cognitive processing score of 6.5 indicates that the individual provided 6.5 words reflecting cognitive processing for every 100 words spoken.

### Analysis

Descriptive and inferential statistics (i.e., *t* tests and chi-square) were used to address all research questions. Independent *t* tests were used to assess group differences in clinical reasoning performance (PEF), linguistic markers of cognitive processing, and perceived mental effort. Chi-square analyses examined group differences in perceived challenges and adaptive inferences (both microanalysis and linguistic analysis). Regarding chi-square tests, given the modest sample size used in this study, the likelihood ratio chi-square was used for the chi-square analysis [58]. An a priori selected *p* value of .05 was used for all inferential analyses, unless otherwise noted.

## Results

The 38 participants were from different specialties (i.e., internal medicine [68.4%), family medicine [13.2%), and surgery [18.4%)) with varying levels of expertise (i.e., intern [42.1%], resident [18.4%], and attending [39.5%]). The majority of the participants were male (65.8%), with an average age of approximately 36 years.

### Clinical reasoning performance

An independent *t* test revealed statistically significant group differences in PEF performance (*t* (36) = 7.22, *p* < .05, Cohen’s *d* = 2.32). Thus, individuals from the live scenario condition (*M* = 0.72, *SD* = 0.07) outperformed those from the video condition (*M* = 0.52, *SD* = 0.10). The effect size for the performance measure is considered very large [59].

### Reflective judgments and cognitive processing

To examine group differences in physicians’ perceived challenges, adaptive inferences, and cognitive processing during the encounter, we used data from SRL microanalytic questions, think-aloud transcripts, and a self-report measure.

### Perceived challenges

Descriptive analysis revealed two categories with sufficient cell sizes to run inferential statistics: *analysis of data* and *lack of case information*. The total frequency counts across groups for the *knowledge/skills* (*n* = 1; 2.6%) and *no challenge* (*n* = 0; 0.0%) categories were negligible (see Table 1). A Bonferroni correction was used to adjust for the two comparisons, resulting in a more conservative *p* value of .025. Statistically, significant group differences emerged for both *analysis of data* (*χ*^*2*^ (1) = 7.16, *p* < .025, *ϕ* = 0.43) and *lack of case information* (*χ*^*2*^ (1) = 5.15, *p* < .025, *ϕ* = 0.36). Thus, statistically significantly more physicians in the live condition (*n* = 15, 78.9%) than video (*n* = 7, 36.8%) focused on the integration and synthesis of data as their primary challenge to accurately diagnose the case. Conversely, a statistically significant greater number of physicians in the video condition (*n* = 8, 42.1%) relative to the live group (*n* = 2, 10.5%) focused their attention on a perceived lack of case information.

**Table 1:** Frequency and percentage of perceived challenge responses across instructional group

Perceived challenge	Video (*n* = 19) *n* (%)	Live (*n* = 19) *n* (%)	Total (*n* = 38) *n* (%)
Knowledge and skill	1 (5.3%)	0 (0%)	1 (2.6%)
Analysis of data	7 (36.8%)	15 (78.9%)	22 (57.9%)
Lack of case information	8 (42.1%)	2 (10.5%)	10 (26.3%)
No/none	0 (0%)	0 (0%)	0 (0%)
Other	3 (15.8%)	3 (15.8%)	6 (15.8%)

The sum percentage for the live condition exceeded 100% given that one participant in this group provided two codeable responses

### Adaptive inferences

Descriptive analysis of the adaptive inference microanalytic question revealed that *clinical tasks* and *none* were the only two response categories with sufficient cell sizes to run inferential statistics (see Table 2). The clinical task category included responses pertaining to key activities of the clinical reasoning process (e.g., history, tests, and physical exam) while the none category reflected physician perceptions that they did not need to change anything to improve performance. Chi-square analyses revealed no statistically significant group differences across either category.

**Table 2:** Frequency and percentage of adaptive inference responses across instructional group

Adaptive inference	Video (*n* = 19) *n* (%)	Live (*n* = 19) *n* (%)	Total (*n* = 38) *n* (%)
Clinical tasks (history, tests, exam)	5 (26.3%)	4 (21.1%)	9 (23.7%)
Process (total)	0 (0%)	2 (10.5%)	2 (5.3%)
- Identify symptoms	0 (0%)	1 (5.3%)	1 (2.6%)
- History and demographics	0 (0%)	1 (5.3%)	1 (2.6%)
- Clarifying/prioritizing symptoms	0 (0%)	0 (0%)	0 (0%)
- Integration	0 (0%)	0 (0%)	0 (0%)
- Comparing/contrasting diagnoses	0 (0%)	0 (0%)	0 (0%)
None	9 (47.4%)	10 (52.6%)	19 (50.0%)
Other	5 (26.3%)	3 (15.8%)	8 (21.1%)

An interesting pattern emerged, however, as part of a follow-up descriptive analysis of aggregated group data for the microanalytic adaptive inference data. Fifty percent (*n* = 19) of the physicians did not believe they would do anything differently to improve their performance. Given the unexpectedly high number of “no change needed” responses, we conducted additional exploratory, post hoc analysis. Specifically, we used expert consensus for performance-based scores from three components of the PEF (i.e., leading diagnosis, supporting evidence, and management of components) to identify physicians who performed at an acceptable or subpar level. *Acceptable* was defined as a score of at least 50% across the three components, while a *subpar* designation involved a score of less than 50% on any of these components. Approximately, 42% (*n* = 8) of the physicians who provided a “no change needed” response exhibited subpar performance; that is, many physicians reported that they did not need to change or improve anything about their clinical reasoning task performance even though they underperformed.

In terms of linguistic analyses of adaptive inference indicators from the think aloud, chi-square analysis revealed a statistically significant group difference (*χ*^*2*^ (1) = 3.81, *p* = .05, *ϕ*= 0.31). Thus, a greater number of physicians in the live scenario condition (*n* = 17; 89.5%) relative to the video condition (*n* = 12; 63.2%) made statements that reflected appraisals of their initial approaches to the case. Examples of these types of adaptive inference markers from the live scenario condition include (i.e., markers in boldface) “But I **never** asked him specifically if he’s ever had a history of a heart attack” (negation) and “I think I **should** have asked him if he was on a statin” (modality).

### Cognitive processing

The LIWC analysis was conducted to examine differences in the cognitive processing of physicians during the reflection activity. Given that the Levene’s test for equality of variances was statistically significant (i.e., unequal group variances), we used Welch’s *t* test to assess group differences. A statistically significant difference was observed (Welch’s *t* (27.7) = 1.97, *p* < .05, *d* = .70), with an effect size approaching large. Individuals from the live scenario condition (*M* = 18.81, *SD* = 1.89) displayed a greater number of words reflective of higher levels of cognitive processing than those from the video condition (*M* = 16.84, *SD* = 3.49). For instance, a live scenario participant stated (boldface words reflect higher-level cognitive processes suggesting increased mental effort) “At this point I am **trying** to tease out **whether or not** this is **something** that is **specifically related** to exercise, if starting and **stopping** starts and stops the pain, **or if** it is something that happens to occur at the same time. **But all** of his answers pushed it towards very much **linked** to the exercise.” In contrast, this video participant used fewer of these causal and differentiating words: “He’s saying it’s burning. It **could** be heartburn. **Seems** like he has a history of heartburn. The pain is similar to that, **but different**. Doesn’t **seem** to be in **any** distress. It woke him up this morning.”

## Discussion

The primary objective of this study was to examine differences in the reflective experiences and clinical reasoning performance of physicians participating in different types of simulation formats. This study is important because it adds to the paucity of studies examining simulation effectiveness and offers a nuanced analysis of the underlying reflective and cognitive processes of physicians during clinical activities. This study also has important implications for learning and practice, specifically the need for educators and trainers to be cognizant of the types of thoughts and reactions medical professionals exhibit when immersed in different learning experiences.

### Differences in clinical reasoning performance

Consistent with expectations, we found that physicians from the live scenario showed stronger clinical reasoning abilities than those in the video condition. Interestingly, although this effect was found to be quite large (Cohen’s *d* = 2.32), this result diverges from prior research showing equivocal results across different learning formats (i.e., paper case, videos, and live scenarios [14, 34]).

These discrepant findings could be partially explained by methodological differences in the studies (e.g., sample and outcomes measure). In prior research, authors often used medical student populations whereas in the current study, we included experienced physicians. Perhaps, more experienced physicians benefit from simulated experiences that afford opportunities for greater autonomy and authentic patient interactions. These environments may allow experienced practitioners to draw upon their extensive knowledge base and engage in deeper forms of case conceptualization and analysis—a premise supported by our other findings regarding reflective judgments and cognitive processing (see next section).

Another important methodological difference involves the level of granularity and task-specificity of the dependent measures. We used a task-specific measure of clinical reasoning performance (i.e., PEF) rather than more broad outcomes, such as the OSCE or essay exam. The PEF was directly linked to the assigned case and patient encounter, whereas other studies focused on outcome measures necessitating the transfer or generalization of skills from the learning situation. Thus, although we clearly cannot use our data to make broad generalizations regarding the effects on simulation on clinical performance, it is does suggest that future research should consider the nature and granularity of the performance measures to assess simulation effects.

### Group differences, physician perceptions, and reflective judgments

Consistent with expectations, we found that the live scenario participants exhibited a more adaptive pattern of judgments and cognitive processes following task performance than video case participants. This pattern was observed across the initial completion of the PEF as well as during the video reflection activity that followed. For example, the majority of physicians from the live scenario condition focused on *data analysis skills* (e.g., integrating symptoms, comparing, and contrasting diagnoses) as their primary challenge when completing the PEF, while the video participants seemed mostly concerned about the adequacy of the case scenario; that is, 42% of the video physicians believed that the case *lacked the necessary information*, even though the experts who created the video purposefully included all of the relevant information to identify the correct diagnosis. One implication of this finding is that when experienced physicians watch videos of a doctor-patient encounter they may not be aware of or notice key pieces of information related to the situation or potential diagnoses. This observation is supported by research showing that physicians often miss key information when viewing videos of patient encounters with contextual factors [17, 60].

The results pertaining to adaptive inferences (i.e., conclusions made regarding how to adapt or change one’s approach to clinical reasoning) were also important. In general, group differences emerged when using linguistic analysis of video think-aloud as part of the reflection activity but not when examining microanalytic data following the initial patient encounter. In terms of microanalysis, the physicians from both groups were asked about what they needed to do to improve or sustain high quality clinical skills immediately after completing the PEF. Although no group differences were observed, remarkably, descriptive analysis showed that 50% of the physicians (regardless of group) reported that changes or modifications were *not needed*. These results align with previous research showing that medical students do not consistently focus on such processes at the outset of a patient encounter and often abandon process-oriented ways of thinking when challenges arise [45, 46].

Conversely, linguistic analysis of think-aloud data revealed important differences in physician reflective judgments and cognitive processes. The live scenario group used more language representing reflection and adaptive-oriented thinking. One implication of this finding is that watching a video of one’s own behavior and performance may lead to greater self-awareness that prompts greater analysis of effective and ineffective actions. Further, it is relevant to note that engaging in live simulations *plus* video self-analysis using a structured think aloud protocol differs from the more typical simulation practice emphasizing instructor-led reflections through a post-simulation debriefing. The apparent advantage of video self-reflection following live scenarios also sheds some light on the debate as to whether video-guided reflection, specifically, is advantageous. Two recent systematic reviews suggest that video-assisted debriefing may promote improvements in learning outcomes, performance, and attitudes, whereas this study places an emphasis on participants’ cognitive processes and metacognitive process [61, 62].

Another important implication of this study involves the importance of using multi-method assessment approaches when targeting complex cognitive or regulatory processes during clinical activities. In our study, we utilized both SRL microanalysis and a think-aloud protocol to examine physician reflective judgments at different aspects of the simulation experience (i.e., initial patient encounter and video self-reflection). The use of multiple measures enabled us to provide a more comprehensive and nuanced account of physician experiences during simulated clinical reasoning. Similarly, we believe that researchers should consider different aspects of clinical activities when conducting these types of process-oriented assessments [44, 63]. Because we conceptualized the simulation in terms of both the patient encounter and video reflection, we were able to draw more nuanced interpretations about the physician experience. Cleary and colleagues recently demonstrated the utility and relevance of a *component analysis* of clinical tasks; medical students varied in the accuracy of their evaluative judgments at different points during a clinical encounter (e.g., patient history and physical exam) [44].

Finally, our results have implications for medical educators, specifically the choices they make regarding the use of simulation or more traditional learning modalities. While video cases can help medical students practice and refine clinical skills in an efficient manner, they may not prompt individuals to focus their attention on refinement and self-reflection regarding the quality of their diagnostic reasoning process. Much more research is needed before definitive implications can be made, but it does appear that if medical educators or supervisors want their students to focus more deeply on how they approach a given case, the use of live scenarios and including both a PEF and a video reflection can help to optimize this objective.

### Limitations and areas of future research

Although these results are informative, there are a few limitations that warrant attention. First, the modest sample size prevented us from including moderator variables in the analyses (e.g., type of diagnosis and experience level). Also, the external validity of this study is limited as it only focused on one diagnosis (stable angina), and we exclusively focused on a task-specific measure of clinical reasoning performance (i.e., PEF). To more definitively conclude whether simulation experiences lead to different performance outcomes, future studies need to include a broader array of performance measures.

Another important limitation is that we did not use SRL microanalysis and think alouds across each component of the simulated experience (i.e., encounter plus PEF and video self-reflection). Thus, it was not possible in this study to identify whether the differences observed in adaptive inference were a function of the type of assessment tool (microanalysis vs. think aloud) or the component of the target activity (initial encounter vs video self-reflection). Finally, because the length of the patient encounter video varied across conditions (i.e., approximately 5 min for video and 15 min for live scenario), it is possible that some of the observed group differences were a function of the live participants having more time interacting with the patient.

## Conclusions

The current study adds to the literature examining the differential effects of live scenario and video simulation approaches and demonstrated the utility of using micro-level, context-specific assessment tools during clinical tasks. Based on our study sample, we found that physicians who engaged in live scenario simulations outperformed and showed a distinct pattern of cognitive reactions and judgments compared to physicians who engaged in video simulations. Our study also underscores the use of multi-method assessment approaches when targeting regulatory and cognitive processes, approaches that consider physical performance, and thinking during different aspects of a clinical encounter.

## Supplementary information

**Additional file 1.** Think aloud protocol warm up.

**Additional file 2.** Coding scheme.

## Data Availability

The datasets used and/or analyzed during the current study are available from the corresponding author on reasonable request.

## Abbreviations

PEF: Post Encounter Form
LIWC: Linguistic Inquiry and Word Count
SRL: Self-regulated learning
OSCE: Objective Structured Clinical Exam
