# Supplementary material for: Effects of live and video simulation on clinical reasoning performance and reflection
Source: Adv Simul (Lond). 2020 Jul 31;5:17. doi: 10.1186/s41077-020-00133-1 (PMC7393892; doi:10.1186/s41077-020-00133-1)
Supplement: Supplementary file 1 — Additional file 1. Think aloud protocol warm up. [file 41077_2020_133_MOESM1_ESM.docx]

**Additional file 1**

**THINK ALOUD PROTOCOL WARM UP**

Welcome and thank you for participating in this study. One part of this study involves a think aloud interview. In the think aloud interview, we will be interested in your thoughts leading to your diagnostic decisions.

We are going to do a warm up activity now to acquaint you with this process. I am going to ask you to “think aloud” as you work through some practice questions. What I mean by think aloud is that *I want you to say your thoughts out loud from the moment you finish hearing the practice question until your final answer. Say as much as you feel comfortable saying. Don’t try to plan or explain what you say, just act as if you were speaking to yourself.* If you are silent for a long time, I’ll ask you to think aloud. Do you understand what I would like you to do?

1. **Name 5 animals that live in the zoo?** (Any other thoughts while you were answering the questions? I want to hear those thoughts too but don’t try to explain them, just think aloud.)
2. **What is the 6^th^ letter after B?** (We don’t want a summary, we want to her your thoughts. Reinforce the utterances that were correct—e.g. saying C,D,E,F,G out loud if they do this).
3. **What is the fifth letter before M?** (Again, reinforce what was done right)
4. **If needed (because participant is having difficulty with concept)-- Describe the last time you had a meal at a restaurant. Describe all the steps involved in getting and consuming the meal until you leave the establishment.**

In the study, after you watch each of 3 videotapes, a research assistant will enter the room and ask you to think aloud while watching the videotape a second time. We want you to describe how you arrived at the diagnosis for the patient in the videotape using this think aloud technique.

**INSTRUCTIONS BEFORE WATCHING VIDEOTAPES**

I am going to ask you to “think aloud” as you watch the videotape again. I want you to describe how you arrived at the diagnosis and treatment for the patient in the videotape. *Please say your thoughts out loud from the moment we start the videotape until your final diagnosis and treatment is reached.* *Say as much as you feel comfortable saying. Don’t try to plan or explain what you say, just act as if you were speaking to yourself.* Any questions? **Begin videotape.**
