# Supplementary material for: Effects of live and video simulation on clinical reasoning performance and reflection
Source: Adv Simul (Lond). 2020 Jul 31;5:17. doi: 10.1186/s41077-020-00133-1 (PMC7393892; doi:10.1186/s41077-020-00133-1)
Supplement: Supplementary file 2 — Additional file 2. Coding scheme. [file 41077_2020_133_MOESM2_ESM.docx]

**Additional file 2**

| **Perceived Challenge Codes** | **Definition** |
| --- | --- |
| Knowledge and skill | Response reflects a lack of prior knowledge, skill, competence, or training regarding the chief complaint, related symptoms, and/or diagnosis in question. |
| Lack of case information | Response reflects the participant perceptions of a lack of patient information. The lack of information may have resulted from not being able to interview the patient, other individuals, administer tests, get test results back in time, etc. |
| Analysis of data | Response reflects difficulty with analyzing aspects of the patient’s history and/or presenting symptoms in trying to come to a differential diagnosis. Response may also refer to sorting between multiple differential diagnoses or being flexible when analyzing the data |
| No/none | Response that indicates participant did not encounter any difficulties or challenges in selecting a diagnostic hypothesis. |
| Other | Response does not fit into any of the categories and/or do not pertain to an aspect of the case scenario. |
| **Adaptive Inference Codes** | **Definition** |
| Clinical task | Response reflects gathering additional or different information as part of the patient history, physical exam, or tests or labs. |
| Clinical reasoning process | Response reflects specific clinical reasoning sub-processes, such as identifying symptoms, background/demographic factors, clarifying/prioritizing symptoms, integration of symptoms, and comparing/contrasting diagnoses. |
| None | Response that indicates that participant would not have done anything differently. |
| Other | Response does not fit into any of the categories and/or do not pertain to an aspect of the case scenario. |
